# Retinoid regulated macrophage cholesterol efflux involves the steroidogenic acute regulatory protein

**DOI:** 10.1016/j.dib.2016.03.055

**Published:** 2016-03-19

**Authors:** Pulak R. Manna

**Affiliations:** Department of Immunology and Molecular Microbiology, Texas Tech University Health Sciences Center, School of Medicine, Lubbock, TX 79430, USA

**Keywords:** StAR, steroidogenic acute regulatory protein, retinoids, retinoic acid (RA) and its derivatives, atRA, all-trans RA, (Bu)_2_cAMP, dibutyryl cAMP, RAR, retinoic acid receptor, RXR, retinoid X receptor, LXR, liver X receptor, ABCA1, ATP-binding cassette transporter A1, SREBP-1c, sterol regulatory element-binding protein 1c, Apo-A1, apolipoprotein A1, Macrophages, Retinoids, StAR, Cholesterol efflux, RAR, RXR, ABCA1

## Abstract

Elimination of excess cholesteryl esters from macrophage-derived foam cells is known to be a key process in limiting plaque stability and progression of atherosclerotic lesions. We have recently demonstrated that regulation of retinoid mediated cholesterol efflux is influenced by liver X receptor (LXR) signaling in mouse macrophages (Manna, P.R. et al., 2015, Biochem. Biophys. Res. Commun., 464:312-317). The data presented in this article evaluate the importance of the steroidogenic acute regulatory protein (StAR) in retinoid mediated macrophage cholesterol efflux. Overexpression of StAR in mouse RAW 264.7 macrophages increased the effects of both all-trans retinoic acid (atRA) and 9-cis RA on cholesterol efflux, suggesting StAR enhances the efficacy of retinoic acid receptor (RAR) and/or retinoid X receptor (RXR) ligands. Additional data revealed that atRA enhances (Bu)_2_cAMP induced StAR and ATP-binding cassette transporter A1 protein levels. Treatment of macrophages transfected with an LXRE reporter plasmid (pLXREx3-Luc) was found to induce the effects of RAR and RXR analogs on LXR activity.

**Specifications Table**

**Table:** 

Subject area	Cell Biology, Biochemistry, Immunology
More specific subject area	Regulation of macrophage cholesterol efflux and its relevance to atherosclerotic cardiovascular disease
Type of data	Figures
How data was acquired	Cell culture, Western blotting, immunohistochemical staining, transfection, determination of cholesterol efflux
Data format	Analyzed
Experimental factors	Retinoid responsive macrophage cholesterol efflux was evaluated in mock (pCMV5)- and pCMV5-StAR-transfected RAW 264.7 macrophages. Localization of StAR and ABCA1 proteins in these macrophages was also determined by immunohistochemical staining. Additionally, the effects of selective RAR (TTNPB) and RXR (SR11233) analogs were assessed in determining LXR activity
Experimental features	Mouse RAW 264.7 macrophages transfected with StAR were labeled with ^3^H-cholesterol in DMEM/F12 media containing 0.1% BSA, treated with retinoids, and determined for cholesterol efflux. Macrophages treated with atRA and/or 9-cis RA were also evaluated for StAR and ABCA1 protein levels. Additionally, macrophages were transiently transfected with the pLXREx3-Luc plasmid for determining the influence of retinoid signaling on LXR activity
Data source location	Texas Tech University Health Sciences Center, Lubbock, TX, USA
Data accessibility	Data presented in this article and are related to [1]

**Value of the Data**

• The data presented in this article provide insights into the involvement of StAR protein in retinoid regulated cholesterol efflux in mouse macrophages.
• An increase in StAR levels effectively enhances retinoid responsive cholesterol efflux in macrophages.
• Retinoid signaling elevates not only StAR and ABCA1 protein levels but also LXR activity in mouse macrophages.
• Retinoids are capable of limiting and/or regressing atherosclerotic cardiovascular disease.
• These findings may inspire research on the pathophysiology of atherosclerosis and relevant cardiovascular diseases in conjunction with retinoid signaling.

## 1 Data

**Fig. 1:**
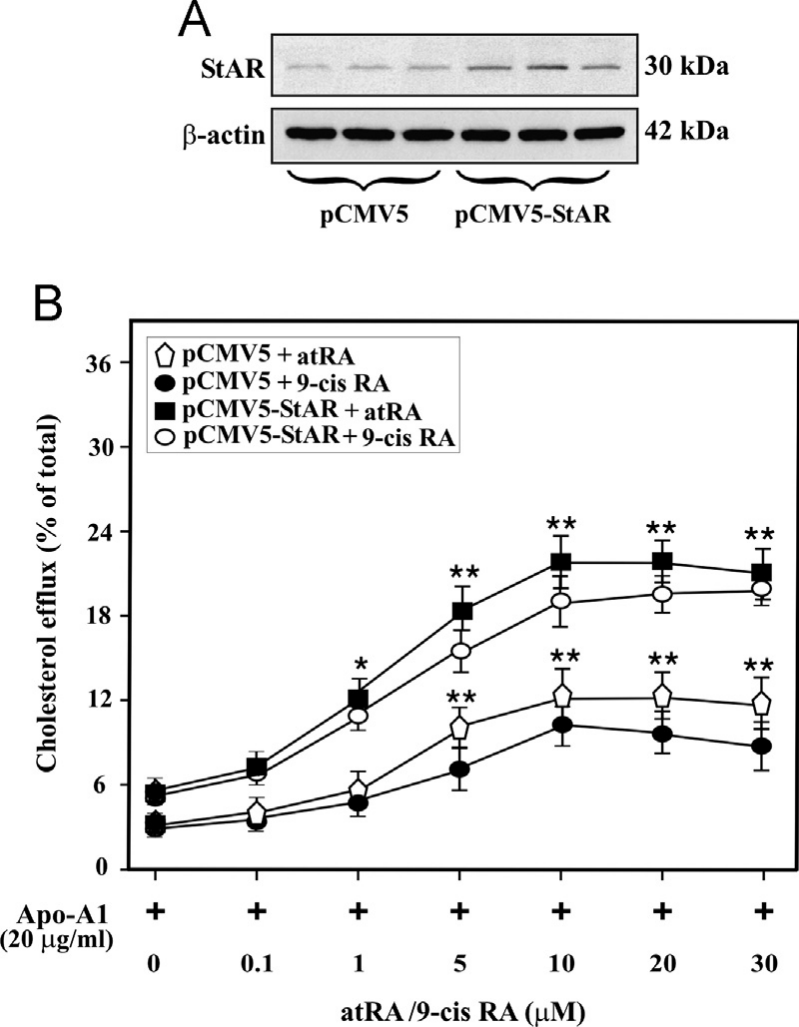
Overexpression of StAR on retinoid responsive macrophage cholesterol efflux. RAW 264.7 macrophages were transiently transfected with either pCMV5 or pCMV5-StAR expression plasmid, as described under Section 2. Representative immunoblots illustrate expression of StAR and β-actin in pCMV5 and pCMV5-StAR transfected groups (A). Following 24 h of transfection, macrophages were labeled with ^3^H-cholesterol for additional 24 h and then treated with increasing doses of either atRA or 9-cis RA (0–30 μM), for 12 h, in the presence of Apo-A1 (20 μg/ml), as indicated. Cholesterol efflux was determined following the assay described under Section 2 (B). Immunoblots shown are representative of 3–5 independent experiments. β-actin expression was assessed as a loading control. Data represent the mean±SE of four independent experiments. *, *p*<0.05; **, *p*<0.01; vs. control.

The data presented in this article show that mouse RAW 264.7 macrophages transfected with the pCMV5-StAR plasmid significantly increased (*p*<0.05) StAR protein expression when compared with mock-transfected (pCMV5) cells (Fig. 1A). Macrophages treated with increasing doses of either atRA or 9-cis RA (0–30 µM), for 12 h, enhanced cholesterol efflux to apolipoprotein A1 (Apo-A1; 20 µg/ml) in a dose dependent manner, over untreated cells (Fig. 1B). Overexpression of StAR in RAW 264.7 macrophages resulted in 2–4 fold increases in cholesterol efflux to Apo-A1 in response to either atRA or 9-cis RA, over the responses seen in mock controls, suggesting StAR plays an important role in retinoid regulated cholesterol efflux in macrophases.

Utilizing immunohistochemical staining, RAW 264.7 macrophages were found to express both StAR and ATP-binding cassette transporter A1 (ABCA1) proteins [1]. Macrophages treated without or with atRA (10 µM) or atRA plus (Bu)_2_cAMP (0.1 mM), for 12 h, resulted increases in StAR and ABCA1 protein levels, over untreated cells. Treatment of atRA enhanced expression of both StAR and ABCA1 protein levels. Whereas (Bu)_2_cAMP had no apparent effects, it markedly increased both StAR and ABCA1 protein expression (data not shown; and Ref. [1]). These data are tightly associated with the research article published recently [1], and reinforce the importance of the StAR protein in retinoid mediated cholesterol efflux in mouse macrophages.

**Fig. 2:**
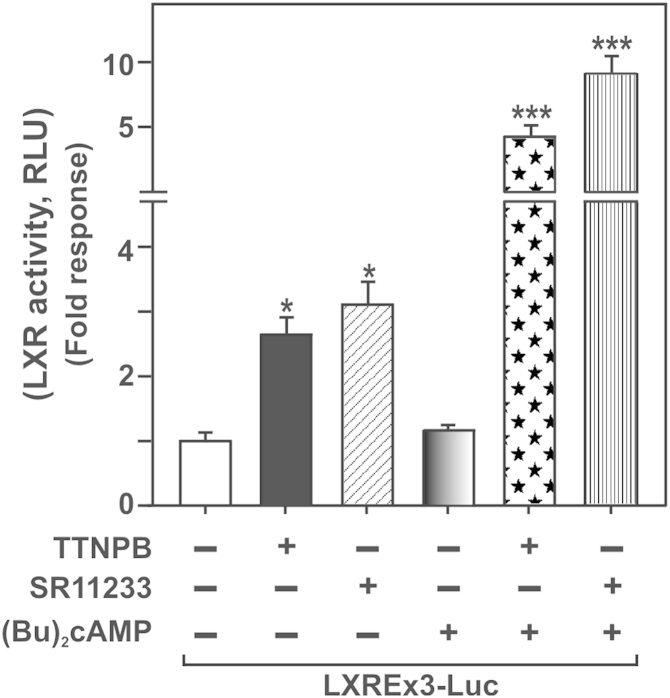
Involvement of retinoid signaling on LXR activity. RAW 264.7 macrophages were transiently transfected with the pLXREx3 reporter plasmid (LXREx3-Luc) in the presence of pRL-SV40, as described under Section 2. Following 36 h of transfection, macrophages were treated without or with TTNPB (5 μM), SR11233 (5 μM), (Bu)_2_cAMP (0.1 mM), or their combination, for an additional 12 h, as indicated. Luciferase activity in the cell lysates was determined and expressed as fold LXR activity (RLU, luciferase/Renilla). Results represent the mean ± SE of 3 independent experiments. *, *p*<0.05; ***, *p*<0.001; vs. control.

We have reported that retinoids, both RAs and retinoic acid receptor (RAR)/retinoid X receptor (RXR) analogs, are capable of enhancing macrophage cholesterol efflux, and these events involve activation of the LXR pathway [1], [2]. Specifically, retinoid mediated macrophage cholesterol efflux has been shown to be influenced by the LXR regulated genes, sterol regulatory element-binding protein 1c (SREBP-1c) and ABCA1. To obtain further insights into the LXR regulatory events, RAW 264.7 macrophages were transfected with an LXRE reporter plasmid (pLXREx3-Luc; [1], [3]), and the effects of selective analogs with affinities to both RAR (TTNPB; Sigma-Aldrich; St. Louis, MO) and RXR (SR11233; Sigma-Aldrich) on luciferase/LXR activity were determined. As illustrated in Fig. 2, LXRE transfected macrophages treated with TTNPB (5 µM; [1]) and SR11233 (5 µM; [1]), individually, resulted in 2.6±0.4 and 3.2±0.6 fold increases in luciferase activity over untreated cells, respectively. LXR activity was unaffected in response to 0.1 mM (Bu)_2_cAMP. Addition of (Bu)_2_cAMP (0.1 mM) to either TTNPB or SR11233 incubation significant enhanced (*p*<0.001) LXR activity, when compared with controls. Co-incubation of SR11233 and (Bu)_2_cAMP displayed ~2 fold increase in LXR activity, over the combined response seen with TTNPB and (Bu)_2_cAMP. These data are connected with our previous findings that demonstrate that both RAs and RAR/RXR analogs effectively enhance macrophage cholesterol efflux through activation of the LXR pathway [1], [2].

## 2 Experimental design, materials and methods

### 2.1 Culture of macrophages, transfections, and luciferase assays

Mouse RAW 264.7 macrophages (ATCC, Manassas, VA) were cultured in DMEM/F12 medium (Invitrogen Life Technologies, Inc., Grand Island, NY) supplemented with 10% FBS, 1% glutamine, and antibiotics (10,000 U/L penicillin and 50 mg/L streptomycin), as described recently [1].

For transfection studies, macrophages were seeded at 2.5×10^5^ cells/well in 6-well plates and transfected using Lipofectamine 2000 reagent (Invitrogen Life Technologies, Inc., Grand Island, NY), under optimized conditions [1], [4], [5]. Full-length mouse StAR cDNA [6] was cloned into the *Eco*RI and *Xba*I sites of the pCMV5 vector, as described previously [1]. LXRE reporter plasmid (pLXREx3; [3]) was obtained from Dr. M.B. Elam (University of Tennessee Health Sciences Center, Memphis, TN). All plasmids were verified by either restriction endonuclease digestion or automated sequencing on a PE Biosystem 310 Genetic Analyzer (Perkin-Elmer, Boston, MA). The amount of DNA used in transfections was equalized with an empty expression vector. Macrophages were transfected with either pCMV5 or pCMV5-StAR expression plasmid (2 µg/well), and following 24 h of transfection macrophages were utilized for experiments.

For luciferase/LXR assay, transfection efficiency was normalized by co-transfecting 10–20 ng of pRL-SV40 vector (Promega Corp., Madison, WI). Luciferase activity in the cell lysates was measured by the Dual-luciferase® reporter assay (Promega) as described previously [7], [8]. Briefly, following treatment, macrophages were washed with 0.01 M phosphate buffered saline (PBS), and 200-250 μl of the reporter lysis buffer was added to the plates. The cellular debris was pelleted by centrifugation at 14,000*g* at 4 °C, and the supernatant was measured for relative light units, RLU (luciferase/renilla) in a TD 20/20 Luminometer (Turner Designs, Sunnyvale, CA).

### 2.2 Cholesterol efflux assay

Cholesterol efflux assay in mouse RAW 264.7 macrophages was determined under optimized conditions [1], [9], [10]. Briefly, macrophages were seeded at 2.5×10^5^ cells/well in 6-well plates 24 h before transfection. Macrophages were transfected with either pCMV5 or pCMV5-StAR using Lipofectamine 2000 reagent (Invitrogen). Following 24 h of transfection, macrophages were labeled with 0.4–0.6 μCi/ml (specific activity 9.25 MBq/mmol) ^3^H-cholesterol (Perkin-Elmer, Boston, MA) for 24 h in DMEM/F12 media containing 0.1% BSA. Cells were then equilibrated in serum free medium containing 0.1% BSA for 4 h. Subsequently, macrophages were washed with 0.01 M PBS and treated without or with increasing doses of either atRA or 9-cis RA (0–30 µM), for 12 h, in the presence of Apo-A1 (20 µg/ml; Sigma-Aldrich). Following treatments, macrophages and media were collected separately. Macrophages were lysed in the lysis buffer (50 mM Tris–HCl, pH 7.6, 150 mM NaCl, 1% NP-40) containing protease inhibitor cocktail (EMD Millipore, Billerica, MA) and counted in a liquid scintillation counter (LS 6500, Beckman). Cholesterol efflux was calculated as the percentage of radioactivity recovered in the media over the total (cells plus media) radioactivity. Cholesterol efflux assays were carried out in triplicates.

### 2.3 Immunohistochemical staining

Immunohistochemical analysis was carried out following procedures described previously [1], [11]. Macrophages were grown (5×10^5^ cells/dish) at 60×15 mm^2^ dishes containing 4–5 coverslips. Following 24 h of seeding, macrophages were treated without or with atRA (10 µM) or atRA plus (Bu)_2_cAMP (0.1 mM) for 12 h. After treatments, macrophages were washed with 0.01 M PBS, fixed with 4% paraformaldehyde, and permeabilized with 0.1% Triton X-100. Macrophages were blocked with 1% BSA-PBS for 20 min and then incubated with StAR [12] and ABCA1 (Millipore, Temecula, CA) for 1 h at room temperature [1]. Following washing, macrophages were incubated with Alexa Fluor 488 and 565 anti-rabbit and anti-mouse secondary antibodies (Invitrogen) in 1% BSA-PBS. The fluorescent images were captured using a Nikon T1-E Eclipse 80i microscope with A1 confocal and a 60X objective, and analyzed using the NIS Elements program (version 3.00 SP7; Nikon, Japan).

### 2.4 Western blot analysis

Immunoblotting studies were carried out using total cellular protein [1], [5], [13], [14]. Following treatments, cells were washed with 0.1 M PBS, harvested in lysis buffer, sonicated, centrifuged at 10,000*g* for 10 min at 4 °C, and supernatant was collected. The protein concentration in the supernatant was measured with Pierce BCA protein assay kit (Thermo Scientific, Rockford, IL). Equal amounts of protein (75-90 µg) were loaded onto 10-12% SDS-PAGE (Bio-Rad Laboratories, Inc., Hercules, CA). After electrophoresis, the proteins were electrophoretically transferred onto Immuno-Blot PVDF membranes (Bio-Rad Laboratories), which were probed with specific antibodies that recognize StAR and β-actin (Applied Biosystems/Ambion, Austin, TX) [1], [12]. Following overnight incubation with primary antibodies, the membranes were washed and incubated with horseradish peroxidase-conjugated secondary antibodies for 1 h at room temperature. The immunodetection of different proteins was determined using a Chemiluminescence Imaging Kit (Perkin-Elmer), and the intensity of bands was evaluated using an image analyzer (Quantity One Software, Bio-Rad Laboratories). Detection of different proteins was assessed using identically processed membranes; however, the same membranes were also analyzed by stripping and reprobing.

### 2.5 Statistical analysis

Statistical analysis was performed by ANOVA using Statview (Abacus Concepts Inc., Berkeley, CA) followed by Fisher׳s protected least significant differences test. All experiments were repeated at least three times. Data presented are the mean ± SE, and p<0.05 was considered significant.
