# Supplementary material for: Retinoid regulated macrophage cholesterol efflux involves the steroidogenic acute regulatory protein
Source: Data Brief. 2016 Mar 19;7:940–5. doi: 10.1016/j.dib.2016.03.055 (PMC4818342; doi:10.1016/j.dib.2016.03.055)
Supplement: Supplementary file 1 — Supplementary material [file mmc1.docx]

**Conflict of Interest**

The author declares no conflict of interest.
